# Intermediate Cu^II^ATCUN Species Reacts With Biomolecules Within Time Windows of Biological Processes

**DOI:** 10.1002/chem.202503280

**Published:** 2025-12-30

**Authors:** Iwona Ufnalska, Wojciech Bal

**Affiliations:** ^1^ Institute of Biochemistry and Biophysics Polish Academy of Sciences Warsaw Poland

**Keywords:** labile copper, copper‐ATCUN complexes, kinetics of competition reactions, properties intermediate species

## Abstract

The ATCUN motif is the N‐terminal peptide/protein sequence Xaa‐Zaa‐His, bearing the N‐terminal amine and Zaa ≠ Pro, which effectively binds some transition metal ions including Cu^II^ in a square‐planar geometry. This motif has attracted much attention, due to its distinctive copper binding properties and abundance in human proteome. However, the inertness and poor reactivity of Cu^II^ATCUN complexes appear at odds with the biological reality of dynamically changing copper balance, which is maintained through redox and trans‐chelation reactions. We have demonstrated a multi‐step character of Cu^2+^ binding to ATCUN sequences, with partially coordinated intermediate species present within time windows spanning from hundreds of milliseconds to seconds. Based on indirect evidence and chemical analogy we previously proposed that these transient species could serve as reactive intermediates in biological copper redox and exchange reactions. Here we used double mixing stopped‐flow experiments to address this issue directly for a model tetrapeptide (DAHK) representing the ATCUN motif at the N‐terminus of Human Serum Albumin. We found that, unlike the final 4‐nitrogen coordinated (4N) square‐planar complex, the transient 2N intermediate readily exchanged Cu^II^ with histidine and was rapidly reduced to Cu^I^ by glutathione. These results pave way to re‐evaluation of physiological roles of ATCUN motifs.

## Graphical abstract

We have demonstrated that a partially coordinated intermediate species formed in a multistep reaction of the Cu(II) ion with a model peptide representing the N‐terminus of Human Serum Albumin exhibits remarkable lability and redox‐activity, which strongly supports its physiological relevance.

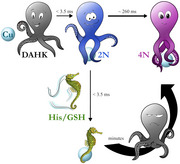

The N‐terminal Xaa‐Zaa‐His peptide sequences, known as Amino‐terminal Copper and Nickel (ATCUN) binding motifs, are ubiquitous in Nature, including the human proteome [1]. These motifs provide high affinity binding sites for Cu^II^, Ni^II^ and some other metal ions prone to forming square‐planar complexes with nitrogen ligands [2, 3]. Two ATCUN sites most prominent for human copper biology are the DAH sequence present in Human Serum Albumin (HSA) and the MDH sequence present in hCtr1 cellular copper transporter. The DAH sequence is often called the N‐terminal site (NTS) in the context of serum albumins [4, 5]. Both these sites exhibit very similar Cu^II^ binding affinities, with *K*_d_ of 0.1 pM at blood physiological pH 7.4 [6, 7]. The current paradigm holds that HSA carries Cu^II^ ions in blood serum, while hCtr1 is supposed to collect and transport them across the membrane into the cell cytosol, upon the reduction to Cu^I^. One could then expect a facile Cu^II^ transfer from HSA to hCtr1. However, when reproduced *in vitro* using the Cu^II^HSA complex and a 14‐peptide modeling the hCtr1 N‐terminus, the equilibration was very slow, with the first‐order reaction half‐times *t*_½_ of 15 min [6]. The same reaction rate was observed for the Cu^II^ transfer from HSA to hepcidin, a peptide hormone bearing the ATCUN (DTH) N‐terminal sequence [8]. Other ATCUN peptides were even more sluggish in similar reactions, with *t*_½_ of the order of hours [9, 10, 11].

Previously, we described a stepwise character of the Cu^2+^ ion reaction with ATCUN peptides. The overall mechanism of this reaction, presented in Scheme 1, features a kinetically metastable intermediate 2N complex, containing the Cu^II^ ion coordinated at the amine and the imidazole nitrogens (IC). The *t*_½_ values for its rearrangement into the final 4N ATCUN complex range from 100 ms for GGH‐COOH, the generic ATCUN peptide, up to several seconds, depending on the peptide sequence and reaction conditions [9, 12, 13, 14]. Based on the spectroscopic similarity between the IC and the complex formed by GGH‐COOH around pH 5, and the 2N coordination mode of the latter, we proposed that IC may be a missing redox‐reactive species, capable of fast Cu^2+^ ion exchange [9]. On this basis we hypothesized that key steps in extracellular copper biochemistry could be conveyed by IC and similar transient, partially coordinated complexes [15, 16]. Here we provide direct experimental evidence supporting this hypothesis, using histidine (His) as a representative exchange agent present in blood serum [17], and reduced glutathione (GSH, γ‐Glu‐Cys‐Gly) as a physiological thiol, capable of reducing Cu^II^ATCUN complexes to Cu^I^/GSH species [18]. Incidentally, both His and GSH were proposed as co‐release ligands for synaptic copper ions [19, 20]. Our experiments were performed on DAHK, a model tetrapeptide representing the N‐terminal ATCUN site of HSA, widely considered to deliver copper to tissues [21]. The choice of DAHK rather than DAH was dictated by the structuring role of the Lys side chain in the complex, by its interaction with the His imidazole ring [22].

**SCHEME 1:**
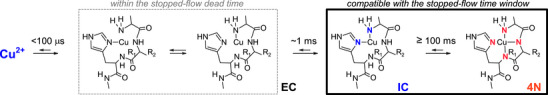
Mechanism of interaction of Cu^2+^ ions with ATCUN sequences; EC (1N)—early complex, IC (2N)—intermediate complex, 4N—thermodynamically stable, final complex [12, 14]. Given the minimal dead times of stopped‐flow technology, typically higher than 1 ms, only the conversion of IC to 4N can be monitored by this technique. The EC was documented previously using the freeze‐quench technique [12].

The reaction of DAHK with Cu^2+^ ions was studied initially in the two‐syringe stopped‐flow setup, illustrated in Figure S2A. The absorption spectra were recorded at 1.5 ms intervals over the observation period of 2 s (Figure 1). The observed reaction is the conversion of IC absorbing at 705 nm, and formed within the instrument dead‐time [12], into the 4N complex. The latter is evidenced by the buildup of its *d‐d* band at 528 nm. The isolated spectra of IC and 4N complexes are presented in Figure S3. The presence of an isosbestic point at 615 nm in this reaction indicates a direct rearrangement of IC into 4N according to Scheme 1. This behavior is common to all simple (e.g. containing one His residue) ATCUN systems studied so far [12, 13, 14]. The inset in Figure 1 provides the corresponding first‐order kinetic fit to the reaction trace at 528 nm. The *k*_obs_ = 3.491 ± 0.008 s^−1^ (*t*_½_ = 198 ± 0.5 ms) was obtained from the global fit of 12 kinetic traces collected in three independent experiments (four runs each). A very low statistical error of *k*_obs_ indicates a high chemical and instrumental reproducibility of the studied system. The length of the IC lifetime provides a time window sufficiently long to conduct three‐syringe double mixing experiments (see Figure S2B) for elucidating the reactivity of IC.

**FIGURE 1:**
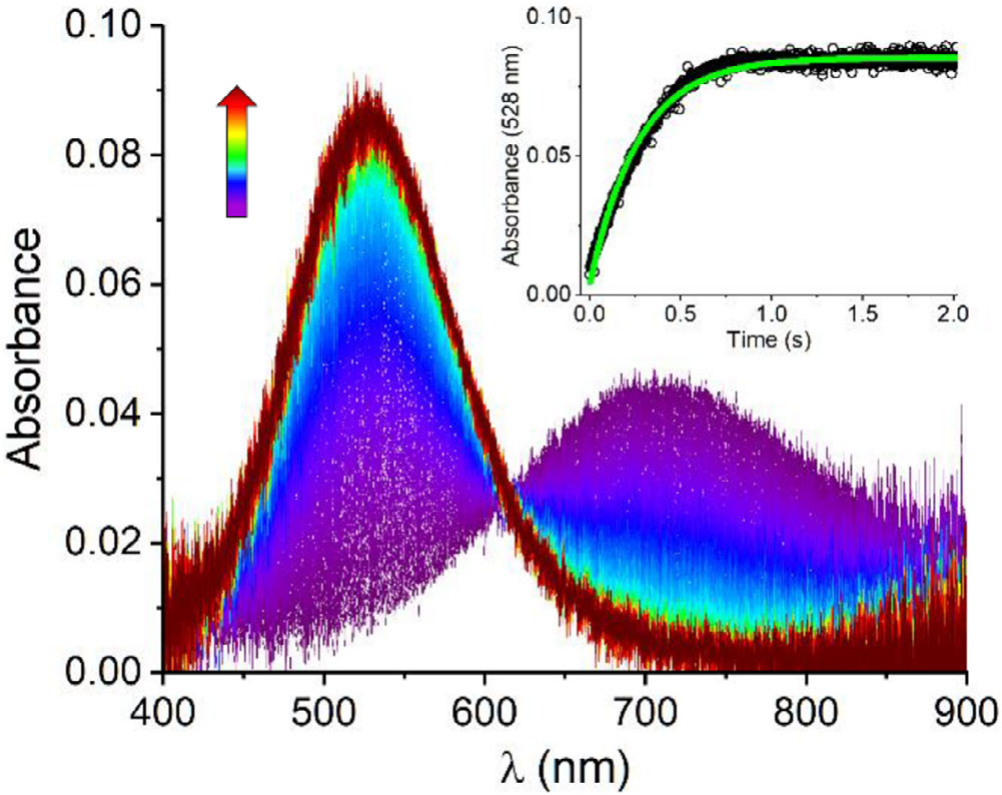
The absorption spectra collected for 2 s with 1.5 ms intervals after mixing of 2 mM DAHK dissolved in 400 mM HEPES buffer with the same volume of 1.8 mM CuCl_2_ dissolved in distilled water, pH 7.4, 25°C. The experiment was performed in a two‐syringe stopped‐flow configuration (Figure S2A). The inset shows the kinetic trace at 528 nm corresponding to the 4N complex formation together with the first‐order kinetic fit to the data.

These experiments were performed in the continuous flow mode, where aqueous Cu^2+^ ions were first mixed with buffered DAHK to create IC. The resulting reaction mixture was mixed with a buffered solution of either His or GSH. As shown in Figure S2B, in the three‐syringe system the solution exiting the first mixer reaches the detector later than the one exiting the second mixer. This time difference (aging time) of the Cu^2+^/DAHK solution was estimated as 4.3 ms, based on the flow rate and the intermixer volume. A control experiment using HEPES alone instead of His/GSH was performed first to confirm the presence of IC at the observation point (Figure S4). No trace of the 4N complex was present in the initial spectrum, as demonstrated in Figure S5A. The first‐order kinetic fit to the reaction trace at 528 nm indicated that the reaction was somewhat slower than in the 2‐syringe system: *k*_obs_ = 2.65 ± 0.02 s^−1^ (*t*_½_ = 262 ±1 ms). However, the direct comparison of kinetic traces obtained in two‐ and three‐syringe setups, shown in Figure S5B, indicates that the actual rate difference between these setups is marginal. We attribute it to instrumental factors, especially as the initial and final states of the reaction were the same for both cases.

The above method of isolating the IC empowered us to test its reactivity with His and GSH and compare it with the reactivity of the 4N complex. The reaction with His was studied first, beginning with establishing the kinetics of Cu^2+^reaction with a two‐fold molar His excess (dictated by the Cu^II^(His)_2_ stoichiometry). This reaction, studied in the two‐syringe setup, occurred within the instrument dead‐time (Figure S6). This was evidenced by the precise overlap of the initial and final spectra, both belonging to the Cu^II^(His)_2_ species absorbing at 645 nm. However, the reaction of the two‐fold His excess with the pre‐equilibrated Cu^II^DAHK complex (i.e. the 4N species) resulted in the slow partition of Cu^II^ ions between Cu^II^DAHK (∼82%) and Cu^II^(His)_2_ (∼18%) (Figure S7). The presence and quantitation of these two complexes was confirmed by the spectral deconvolution, presented in Figure S8A. The equilibration of the system took several minutes, and proceeded according to the first‐order kinetics.

The reverse Cu^II^ transfer, from Cu^II^(His)_2_ to Cu^II^DAHK (Figure S9), proceeded according to the second‐order kinetics, approaching equilibrium after ca. 5 min. of incubation. The equilibrium partition of Cu^II^ ions between Cu^II^DAHK (∼83%) and Cu^II^(His)_2_ (∼17%) was determined by deconvolution of the final spectrum of reaction mixture (Figure S8B). These experimentally determined Cu^II^DAHK/Cu^II^(His)_2_ equilibria agree perfectly well with calculations using the published protonation and Cu^II^ binding constants of DAHK and His. The obtained ratio was 84%/16%, with uncertainty about ±1%, stemming from standard deviations on the constants [5, 23].

The difference in the observed reaction orders and timescales indicates distinct rate‐limiting steps. The Cu^II^ transfer from Cu^II^DAHK to His requires a partial dissociation of the tightly bound tetradentate ligand, most likely via IC, followed by coordination of His in a ternary species, and final replacement of DAHK by the second His molecule. Since His chelates Cu^2+^ ions much faster than DAHK, the dissociation of the Cu^II^DAHK complex is expected to be the rate‐limiting step here. This is consistent with the observed pseudo‐first‐order kinetics. In contrast, the second‐order character of metal ion exchange between the Cu^II^(His)_2_ and Cu^II^DAHK complexes indicates an associative process, in which DAHK attacks Cu^II^(His)_2_. The faster Cu^2+^ transfer from Cu^II^(His)_2_ to DAHK is consistent with the overall properties of these coordination systems, where the higher stability constant of Cu^II^DAHK, combined with its notably smaller *k*_on_ relative to Cu^II^(His)_2_, implies a very small *k*_off_. Nevertheless, it should be emphasized that the observed reaction order can be influenced by equilibria in preceding fast steps and therefore must be interpreted with caution [12].

]In a three‐syringe stopped‐flow experiment, however, where the IC was mixed with the His solution, the outcome was determined by the selected time window of evolution of the Cu^II^/DAHK reaction (Figure S10). The first recorded spectrum already belonged to the Cu^II^(His)_2_ complex (Figure S10A). This means that at just the twofold excess of His over DAHK, all IC‐bound Cu^II^ ions were extracted by His within the 3.5 ms dead time of the instrument. Therefore, unlike the 4N complex, the 2N‐coordinated IC exhibited prominent lability, making it a feasible Cu^II^ delivering agent (Figure 2, left panels). The downstream Cu^II^ transfer driven by the thermodynamic gradient, from His back to the 4N ATCUN complex, took more than one minute (Figure 2, Figure S10B). The reaction rate constant calculated from the stopped‐flow experiment described above, closely matches the one obtained on a conventional spectrophotometer with manual sample mixing (^sp^ *k* = 1.42 ± 0.02 dm^3^·mol^−1^·s^−1^ *vs*.

**FIGURE 2:**
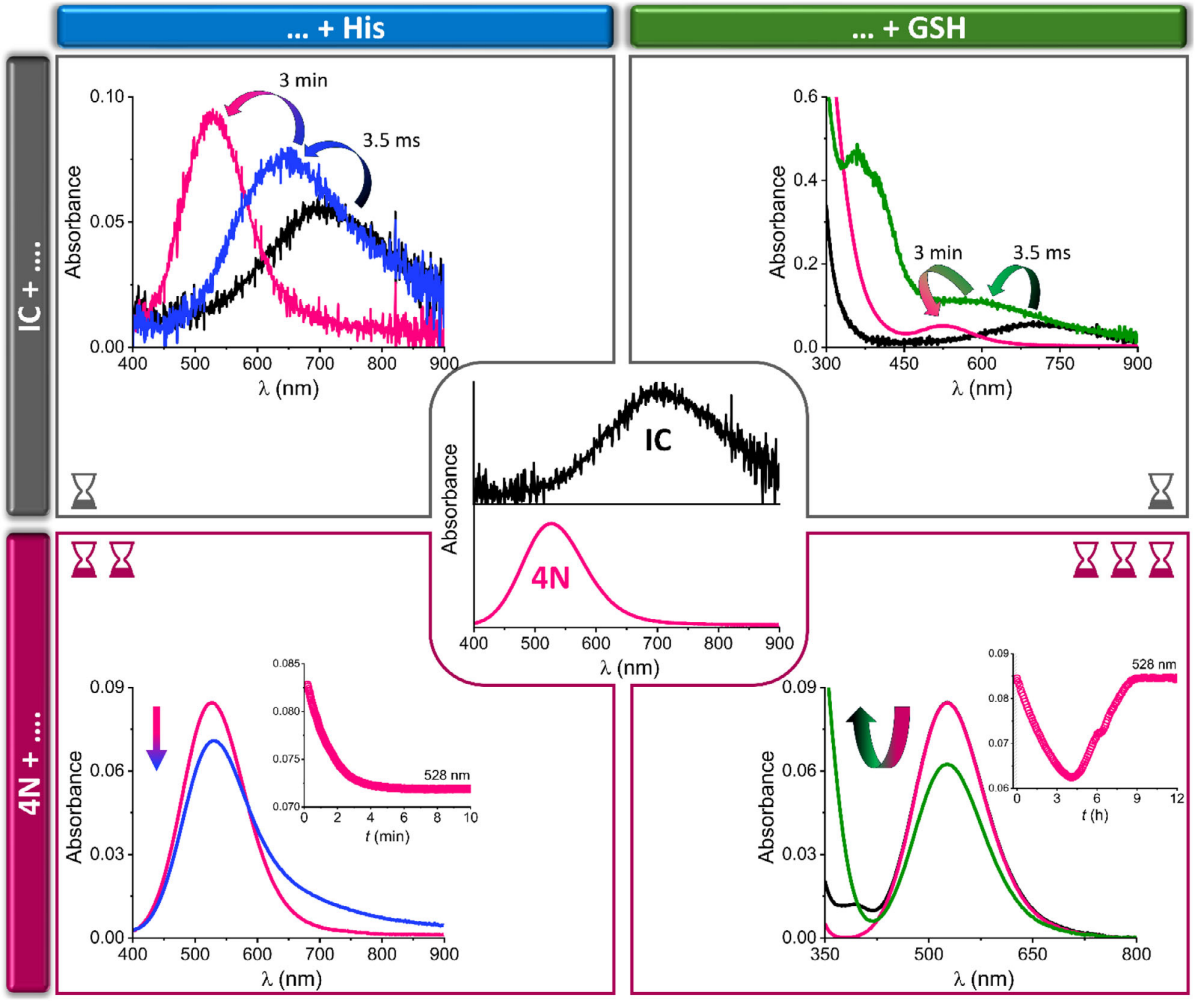
The absorption spectra obtained in a reaction of IC (top panels) and steady‐state Cu^II^DAHK complex (bottom panels) with His (left panels) and GSH (right panels). The reference spectra of IC and 4N complexes are depicted in the central part of the figure. Final concentrations of reagents were: 1 mM DAHK, 0.9 mM CuCl_2_, 2 mM His, 1 mM GSH, and 200 mM HEPES, pH 7.4. Top panels (three‐syringe stopped‐flow experiments): comparisons of absorption spectra of IC transients (black line) with the initial (3.5 ms) and final (3 min) spectra obtained for reactions of IC with His/GSH (blue/green line) (see Figures S10 and S12 for more details). Bottom panels (conventional UV‐Vis spectrophotometer): comparisons of absorption spectra of the 4N complex before (pink line) and after reactions with His/GSH (blue/black line) under steady state conditions. The green line reflects the yield of reduction in the presence of GSH (bottom right); insets show kinetic traces at 528 nm reflecting the 4N complex decay/recovery (see Figures S7 and S9 for further details). A perfect recovery of the Cu^II^DAHK complex after the GSH reoxidation resulted in the overlap of pink and black lines in the bottom right panel. The hourglasses in the corners represent different timescales of examined processes: milliseconds‐to‐seconds (one hourglass), minutes (two hourglasses), hours (three hourglasses).

^s‐f^ *k* = 1.339 ± 0.004 dm^3^·mol^−1^·s^−1^), indicating the consistency of these two approaches. Generally speaking, the transfer of Cu^II^ from Cu^II^IC to Cu^II^(His)_2_ is at least four‐five orders of magnitude faster than that from the 4N complex. One should note here that physiological concentrations of HSA (∼630 µM) [24], His (∼100 µM) [25] and exchangeable Cu^II^ ions (∼4 µM) [17] are much lower than concentrations applied in the above experiments due to experimental requirements. Considering a strong concentration dependence of second‐order kinetics, the lifetimes of Cu^II^(His)_2_ and similar small complexes in the bloodstream can be substantially long [16]. Our finding also strengthens the argument put forward by Kirsipuu et al. regarding the specific role of histidine in Cu^2+^ handling in blood [17]. We would also like to note that the minutes time scale of equilibration in the Cu^II^/DAHK/His system may contribute to the apparent discrepancy between the relatively high abundance of low molecular weight pool of blood serum copper and its much lower abundance proposed based on equilibrium calculations.

Redox properties of the IC were examined in the reaction with GSH, where the cysteine sulfhydryl group served as the electron donor moiety. Full reduction of Cu^II^ to Cu^I^ in the binary Cu^II^/GSH system occurred within the instrument dead time, leading to the formation of a short‐lived Cu^I^ species absorbing at 360 nm and some less populated intermediates (Figure S11) [26]. The immediate Cu^II^ reduction to Cu^I^ was a key feature of this reaction. Mixing of the GSH solution with IC generated *in situ* in the three‐syringe stopped‐flow setup described above yielded an analogous reaction, featuring the Cu^II^ reduction to Cu^I^ within the dead time (Figure S11 vs. Figure S12). In the broader time window the slow appearance of the 4N complex was due to Cu^I^ reoxidation to Cu^II^ by air entering the stopped‐flow system, followed by the Cu^II^DAHK complex formation (Figure S12B). In contrast, only a partial reduction of the 4N complex of Cu^II^DAHK at the same GSH concentration occurred. This process took about 4 h (Figure S13). Therefore, the reduction of Cu^II^IC to a Cu^I^/GSH complex was at least six orders of magnitude faster than the reaction involving the 4N complex. The millisecond timescale of IC reduction by GSH aligns better with the physiological time windows than the hours‐long reduction of the 4N complex, as it provides a basal mechanism for cellular copper uptake, which is known to require Cu^I^ rather than Cu^II^ species [27].

In conclusion, the discovery of 2N kinetic intermediate (IC) in the reaction of Cu^II^ ions with ATCUN peptides paved the way for further studies of kinetics and molecular mechanisms of copper transfers. Here we directly demonstrated that, unlike the fully coordinated 4N, the IC is indeed an active copper‐delivery agent. Its 2N coordination environment fosters both a rapid Cu^II^ ion exchange, as evidenced by reactions with histidine, and a reversible switch between Cu^II^/Cu^I^ redox states in the presence of the GSH/O_2_ redox couple. We therefore demonstrated that the IC, present for hundreds of milliseconds between acquiring the Cu^II^ ion by an ATCUN motif and sequestering it in the 4N complex, can distribute it to other carriers. The time window for the Cu^II^ ion delivery to those carriers is controlled by the first‐order reaction of 4N species formation, and so it depends primarily on the peptide/protein sequence/structure, rather than its concentration. A set of ATCUN peptides/proteins can thus collectively create a versatile pool of reactive Cu^II^ and Cu^I^ species. The mixed first‐order/second‐order kinetics of the copper acquisition and release reactions offers new ways of regulation of biological copper transfers, with His and other small molecules providing delay loops for targeted copper handling and delivery. The 4N ATCUN species thought so far to be biologically active complexes emerge as merely temporary copper storage sites.

## Conflicts of Interest

There are no conflicts of interest to declare.

## Supporting information




**Supporting File 1**: chem70636‐sup‐0001‐SuppMat.docx
