# Supplementary material for: Intermediate CuIIATCUN Species Reacts With Biomolecules Within Time Windows of Biological Processes
Source: Chemistry. 2025 Dec 30;32(8):e03280. doi: 10.1002/chem.202503280 (PMC12929937; doi:10.1002/chem.202503280)
Supplement: Supplementary file 1 — Supporting File 1: chem70636‐sup‐0001‐SuppMat.docx [file CHEM-32-e03280-s001.docx]

Supporting Information

Time window as a determinant of reactivity of Cu^II^ATCUN complexes: interactions of the intermediate complex and their implications for copper physiology

Iwona Ufnalska,* and Wojciech Bal*

Institute of Biochemistry and Biophysics, Polish Academy of Sciences
ul. Pawińskiego 5a, 02-106 Warsaw, Poland

***Materials*.** Reduced glutathione (G4251), 4-(2-Hydroxyethyl)piperazine-1-ethanesulfonic acid sodium salt (HEPES buffer, H3375) and L-histidine (H8000) were purchased from Merck, 99.999% pure copper(II) chloride hydrate (AB202069) was purchased from Linegal Chemicals. The Asp-Ala-His-Lys-NH_2_ (DAHK) peptide was synthesized according to solid phase Fmoc strategy,^[28]^ on a Liberty BlueTM Automated Microwave Peptide Synthesizer (CEM Corporation) on the TentaGel S RAM resin. The obtained crude peptide was purified by high-performance liquid chromatography (HPLC; Knauer) with UV−Vis detection at 220 nm on a C18 Eurospher II column (Knauer). The peptide purity was checked using electrospray ionization mass spectrometry (ESI-MS; Premier, Waters).

***Determination of DAHK concentration***. Concentration of DAHK stock solution was determined by spectrophotometric titration using Cu^2+^ ions. The experiment was performed at room temperature in
1­-cm-path-length quartz cuvette (Hellma) on a Varian Cary 50 Bio spectrophotometer. Constant pH was maintained by 50 mM HEPES (pH 7.4). Figure S1 presents the UV-vis absorption spectra, and the corresponding titration curves obtained at several wavelengths thus providing more reliable peptide concentration assessment (since light scattering is wavelength-dependent). The equivalence point was determined from the intersection of two linear functions common to all plotted curves (global fit).

***Stopped-flow experiments.*** The kinetics of the examined reactions was followed at 25 °C on a
SFM-300 diode-array stopped-flow apparatus (BioLogic) equipped with a 1.0-cm-path-length quartz cuvette. In Cu^2+^ ion binding studies 150 µL of 2 mM DAHK/4 mM His/10 mM GSH dissolved in 400 mM HEPES buffer was mixed with 150 µL of 1.8 mM CuCl_2_ (0.1 mM in the case of GSH) at the 1:1 volume ratio (Figure S2A). In competition experiments 150 µL of 3 mM DAHK dissolved in 400 mM HEPES buffer was first mixed with 150 µL of 2.7 mM CuCl_2_, and then the obtained reaction mixture was further reacted with 150 µL of 6 mM His or 3 mM GSH dissolved in 200 mM HEPES (Figure S2B). Final concentrations of the reagents were as follows: 1 mM DAHK, 0.9 mM or 0.05 mM CuCl_2_, and 2 mM His or 1 mM GSH. The time needed for the solution to reach the observation point after the final mixing in the second mixer^-^ was 2 ms. The shortest data acquisition time of the diode-array detector was 1.5 ms, resulting in the time gap of at least 3.5 ms in the monitored reactions (dead time). Depending on the studied system different time intervals were applied, as given in the figure caption (always being a multiple of the integration time equal to 1.5 ms). Datasets of at least 1000 spectra were collected for each run.

The data were analysed using Origin Pro 2024. The reaction rate constants were determined by fitting the rate laws corresponding to different reaction models to experimental datapoints extracted from the spectra at selected wavelengths, which yielded reaction rate constants *k* and half times *t*_½_. Values calculated for the reaction of Cu^2+^ binding to DAHK in a two-syringe setup were obtained from the global fit of four subsequent kinetic runs from each of three independent experiments, while for the same reaction carried out in a three-syringe setup three consecutive runs were used.

***Reference measurements.*** Experiments on thermodynamically stable species were performed at 25°C in a 1-cm quartz cuvette (Hellma) using a Cary 3500 UV–Vis spectrophotometer equipped with an
air-cooled Peltier system (Agilent Technologies). To ensure comparability with the stopped-flow experiments, reactions were carried out under identical conditions: 1 mM DAHK, 2 mM His, and 1 mM GSH were prepared in 200 mM HEPES buffer, pH 7.4, while 0.9 mM CuCl₂ aliquot was introduced in unbuffered stock solution.

Ligand exchange reactions (Figures 7B and 9B) were monitored at two selected wavelengths, 528 nm and 645 nm, corresponding to the absorption maxima of the 4N Cu^II^DAHK and Cu^II^(His)₂ complexes, respectively. Absorption spectra obtained for the reaction of Cu^II^DAHK with GSH were recorded every 5 min over the 250–800 nm range at a scan rate of 200 nm/min. The kinetic traces shown in Figure S13B account for the actual acquisition times corresponding to each selected wavelength.

In order to quantify the relative contributions of the Cu^II^DAHK and Cu^II^(His)₂ complexes in their reaction mixture (Figure S8) deconvolution of the spectra was performed. The fraction of the 4N complex was estimated by scaling its reference spectrum until it reproduced the left shoulder (dotted line) of the deconvoluted spectrum (pink line). Then, the difference between these two spectra (blue line) was fitted with the recalculated reference spectrum of Cu^II^(His)₂ (dash-dotted line).

**Supplementary figures:**


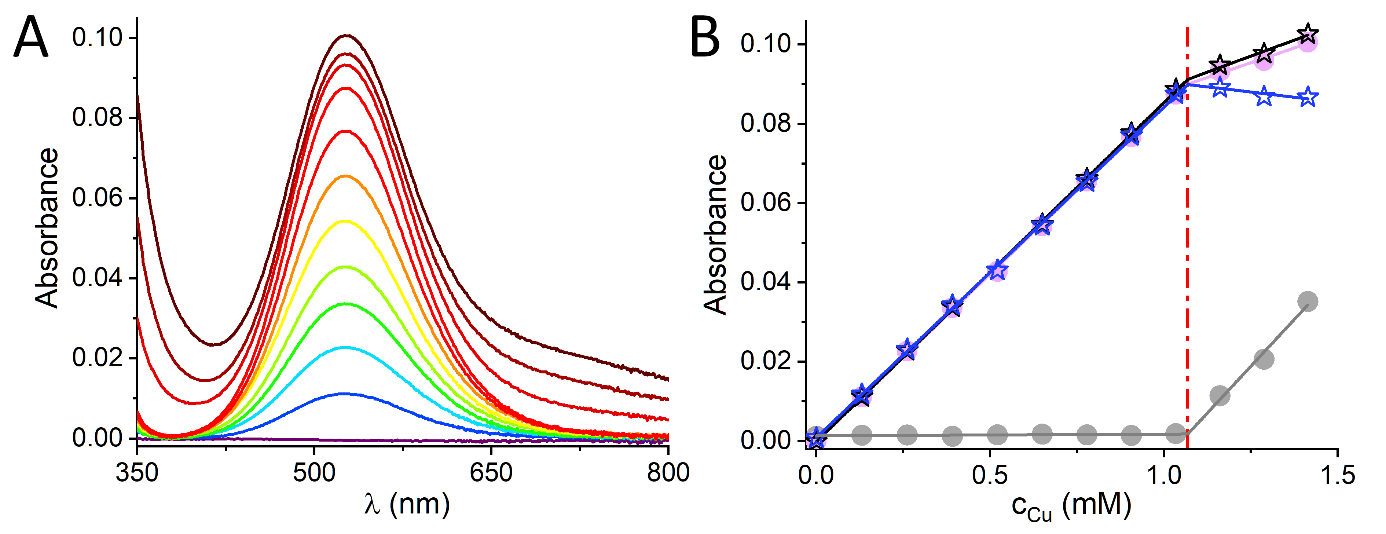


**Figure S1.** (A) UV-Vis spectra illustrating the spectrophotometric titration of ~1 mM DAHK using 1.5 µL aliquots of 70 mM CuCl_2_, pH 7.4, 50 mM HEPES buffer, room temperature; panel (B) represents the method of determining the endpoint of the titration, pink circles – absorbance changes at 525 nm, grey circles – absorbance changes at 380 nm, black stars – absorbance changes at 525 nm corrected by the sample dilution along the titration procedure, blue stars – difference between absorption at 525 nm and 800 nm.


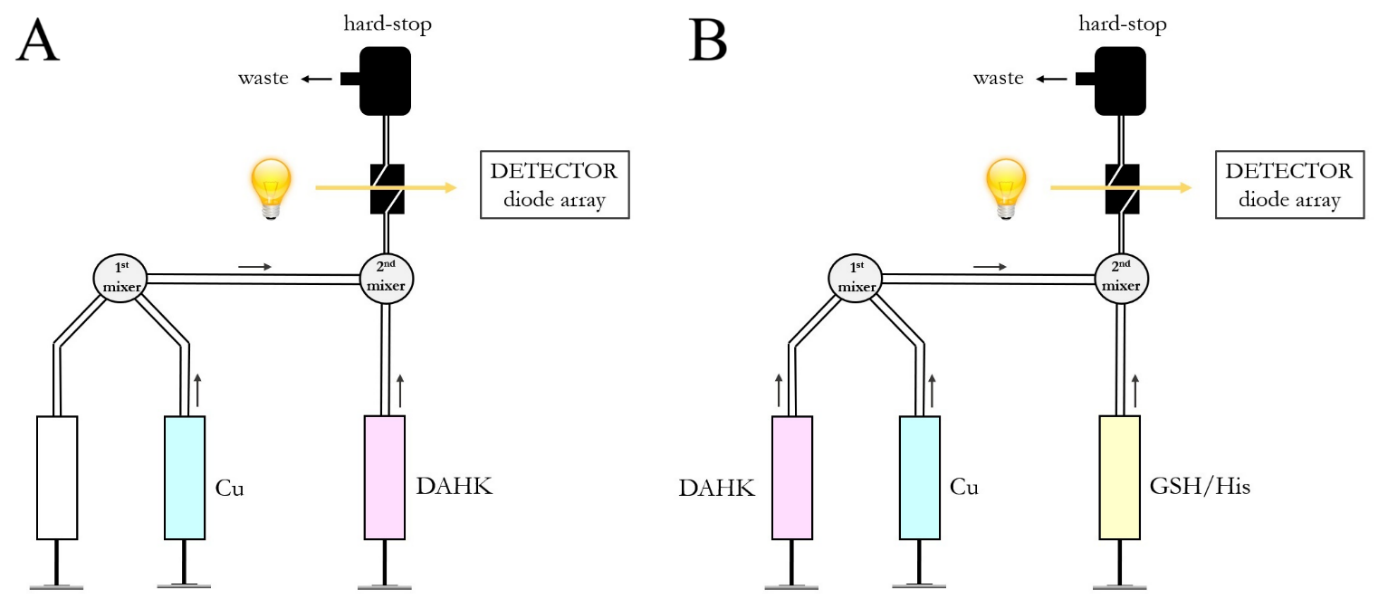


**Figure S2.** Schematic representation of two different stopped-flow configurations applied in the conducted studies: (A) two-syringe setup and (B) tree-syringe setup. In the two-syringe system the reaction of peptide with Cu^2+^ ions is monitored with a 2 ms delay which corresponds to the time required for the solution leaving the second mixer to reach the observation point in the cuvette. In the three-syringe system three reagents are mixed sequentially (there are two mixing events). In the described example DAHK is first mixed with Cu^2+^ ions (mixer 1), leading to the intermediate species formation further reacted with His or GSH (mixer 2). All experiments were carried out in a continuous flow mode (without incubation of the solution between the mixers) yielding the approximately 4.3 ms ageing of the transients, followed by the analogous 2 ms gap in the kinetics of the second reaction.


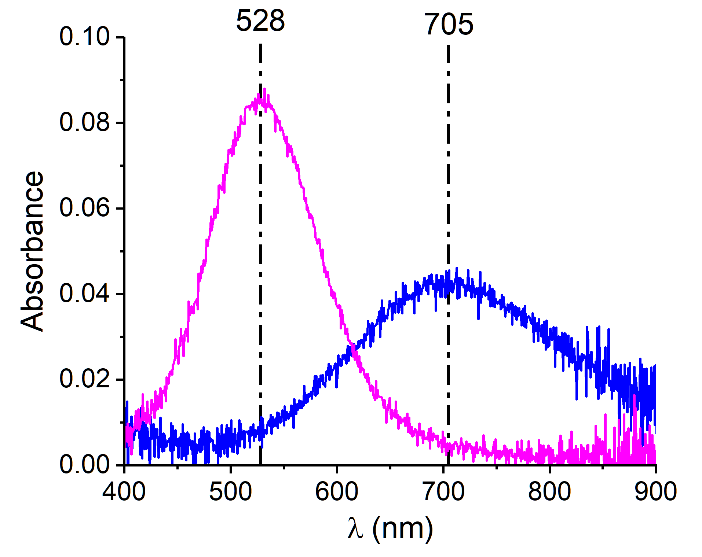


**Figure S3.** The initial (3.5 ms, blue line) and final (2 s, pink line) absorption spectra recorded at 25°C for the reaction of 150 µL of 2 mM DAHK dissolved in 400 mM HEPES buffer, pH 7.4, with the same volume of 1.8 mM CuCl_2_ dissolved in distilled water. The experiment was performed in a two-syringe stopped-flow configuration with 1.5 ms sampling interval (Figure S2A).


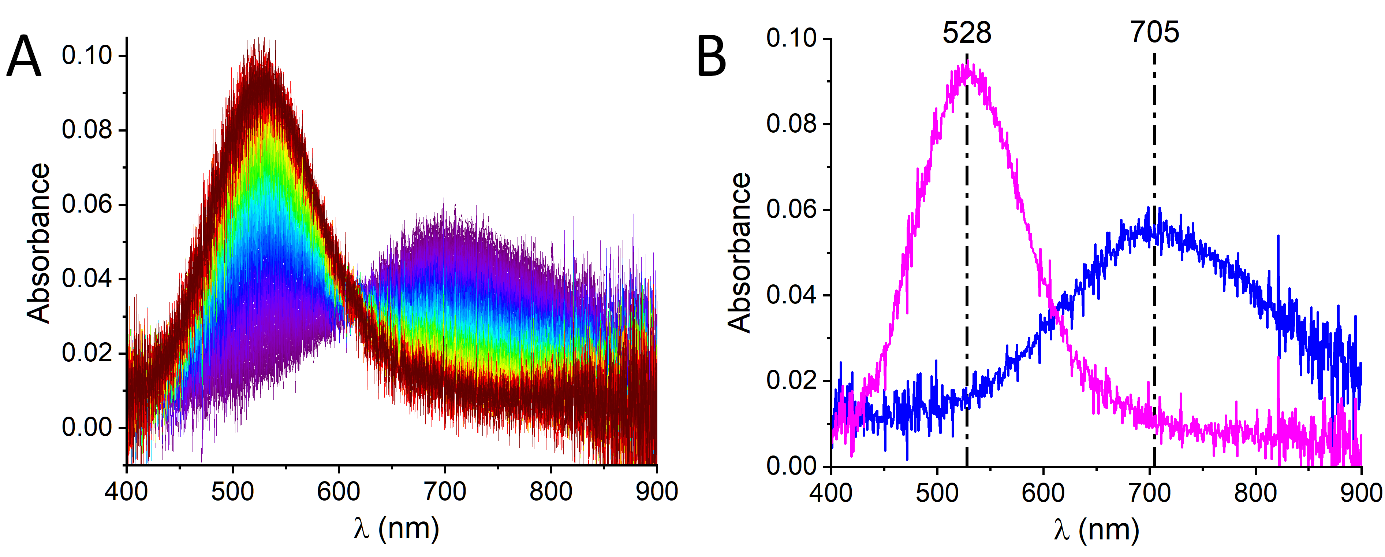


**Figure S4.** The absorption spectra collected for 1.5 s with 1.5 ms intervals after mixing of 150 µL of 3 mM DAHK dissolved in 400 mM HEPES buffer with 150 µL of 2.7 mM CuCl_2_ dissolved in distilled water and 150 µL of 200 mM HEPES, pH 7.4, 25°C. The experiment was performed in a three-syringe stopped-flow configuration (Figure S2B).


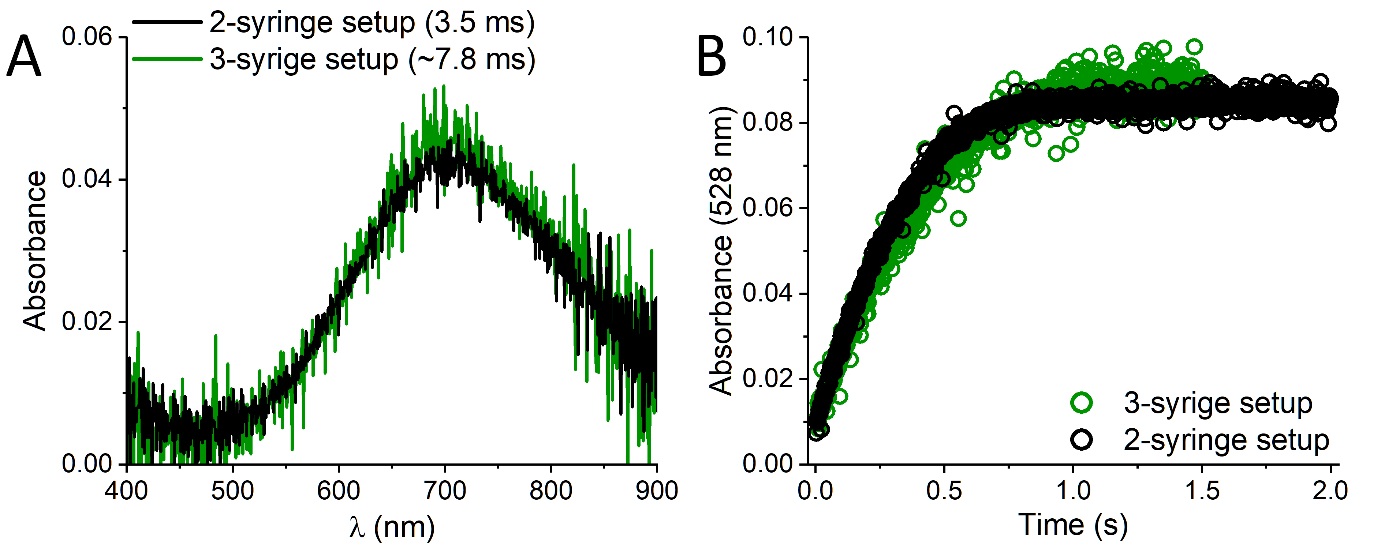


**Figure S5.** Comparison of (A) initial absorption spectra and (B) kinetic traces at 528 nm corresponding to the 4N complex formation obtained for the reaction of DAHK dissolved in 400 mM HEPES buffer with CuCl_2_ dissolved in distilled water in a two-syringe (black line/circles) and three-syringe (green line/circles) experiments in the continuous flow mode; pH 7.4, 25°C, 1.5 ms sampling interval.


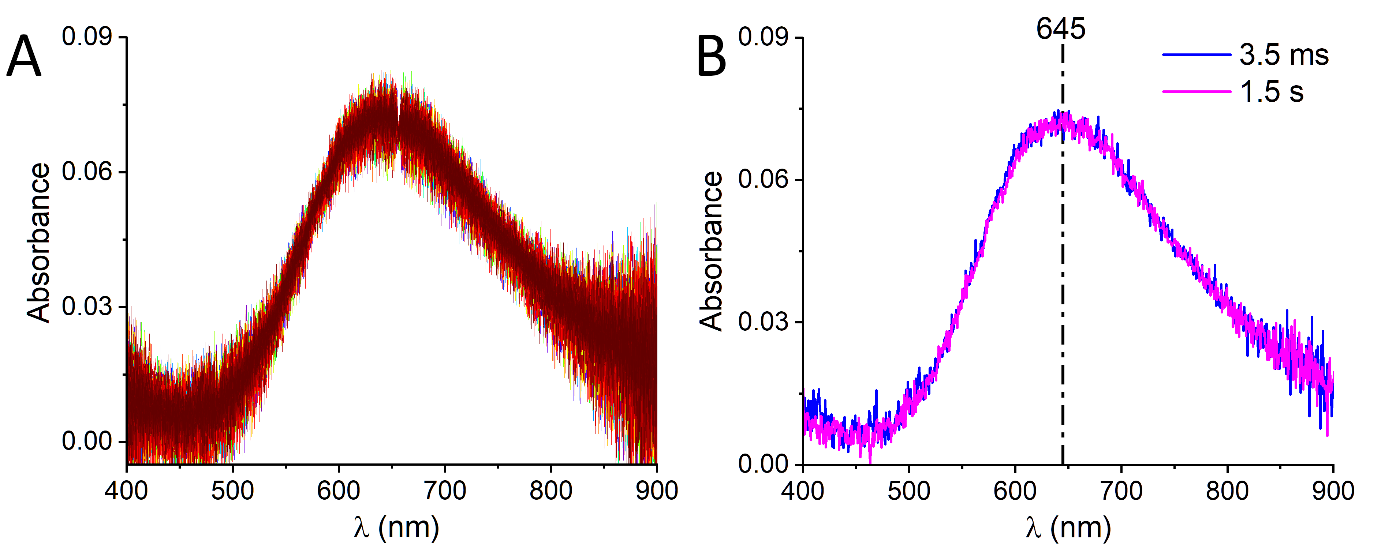


**Figure S6.** (A) The absorption spectra collected for 1.5 s with 1.5 ms intervals after mixing of 150 µL of 4 mM His dissolved in 400 mM HEPES buffer with the same volume of 1.8 mM CuCl_2_ dissolved in distilled water, pH 7.4, 25°C; (B) comparison of the initial (3.5 ms, blue line) and final (1.5 s, pink line) absorption spectra; the experiment performed in a two-syringe stopped-flow configuration (Figure S2A).


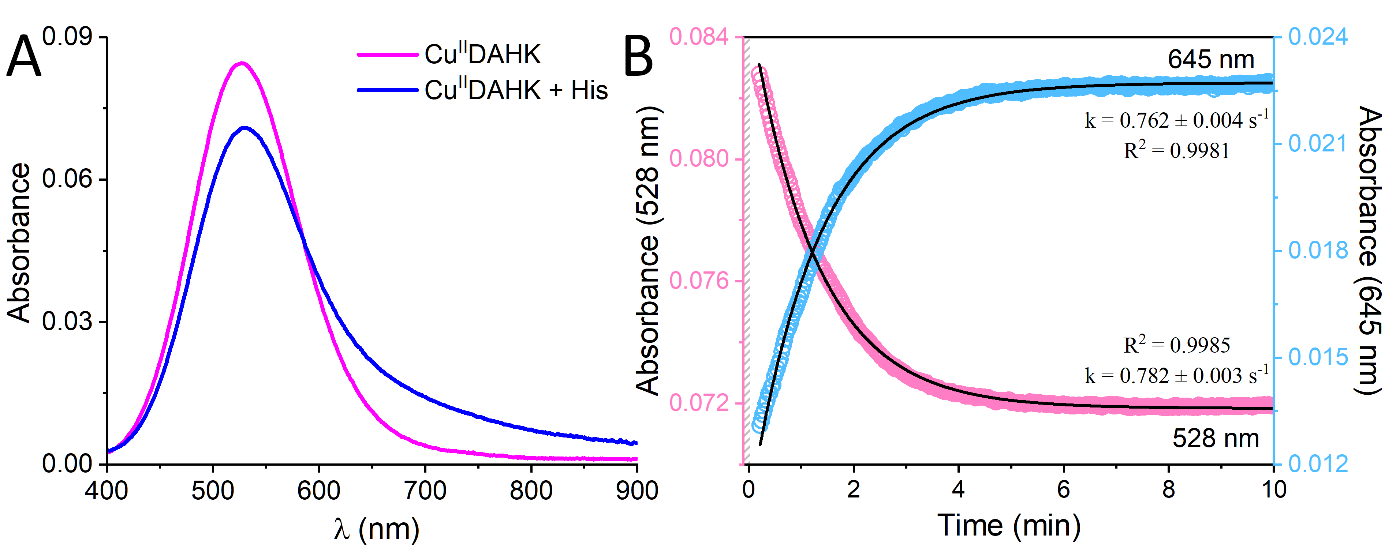


**Figure S7.** (A) Comparison of the absorption spectra obtained under equilibrium for 1 mM DAHK in a presence of 0.9 mM CuCl_2_ before (pink line) and after (blue line) adding of 2 mM His; (B) kinetic traces corresponding to partial transfer of Cu^II^ bound to DAHK (528 nm) to His (645 nm) together with the
first-order kinetic fits, 200 mM HEPES, pH 7.4, 25°C. The experiment was performed on a classic
UV-Vis spectrophotometer with temperature control (Cary 3500).


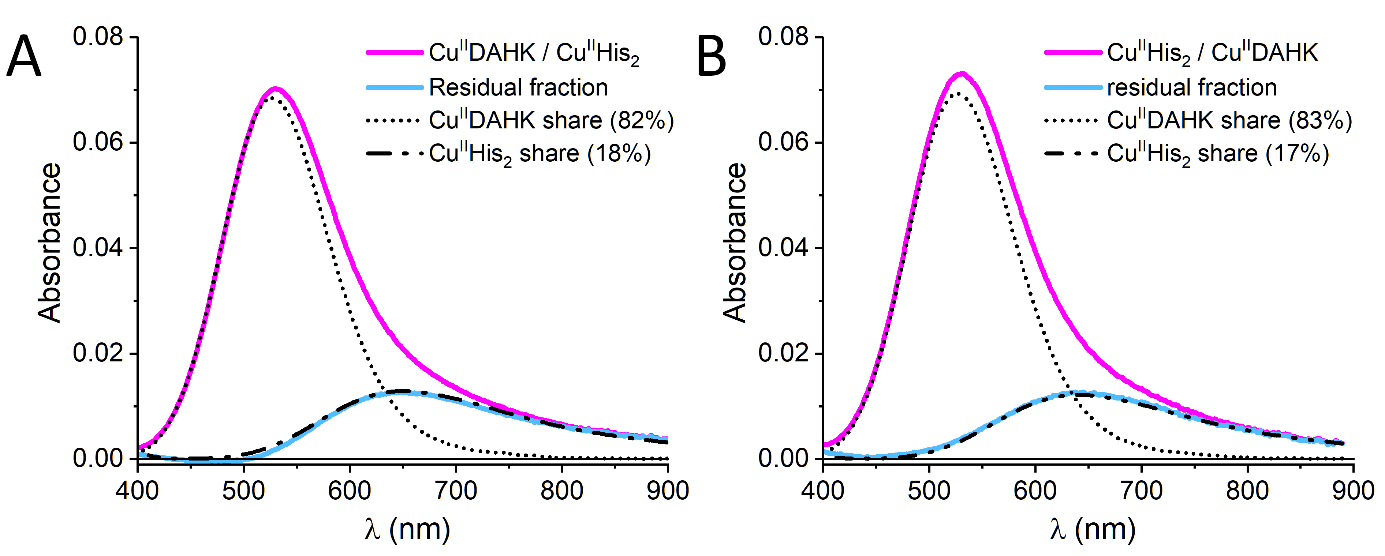


**Figure S8.** Deconvolution of the spectra obtained in a reaction of (A) 1 mM DAHK in a presence of 0.9 mM CuCl_2_ after adding of 2 mM His and (B) 2 mM His in a presence of 0.9 mM CuCl_2_ after adding of 1 mM DAHK, performed on a conventional UV-Vis spectrophotometer (Cary 3500) at 25°C, 200 mM HEPES, pH 7.4; deconvoluted spectrum – pink line, estimated share of 4N complex of Cu^II^DAHK – dotted line, difference between original spectrum and calculated 4N complex share – blue line, estimated share of Cu^II^(His)_2_ – dash dotted line.


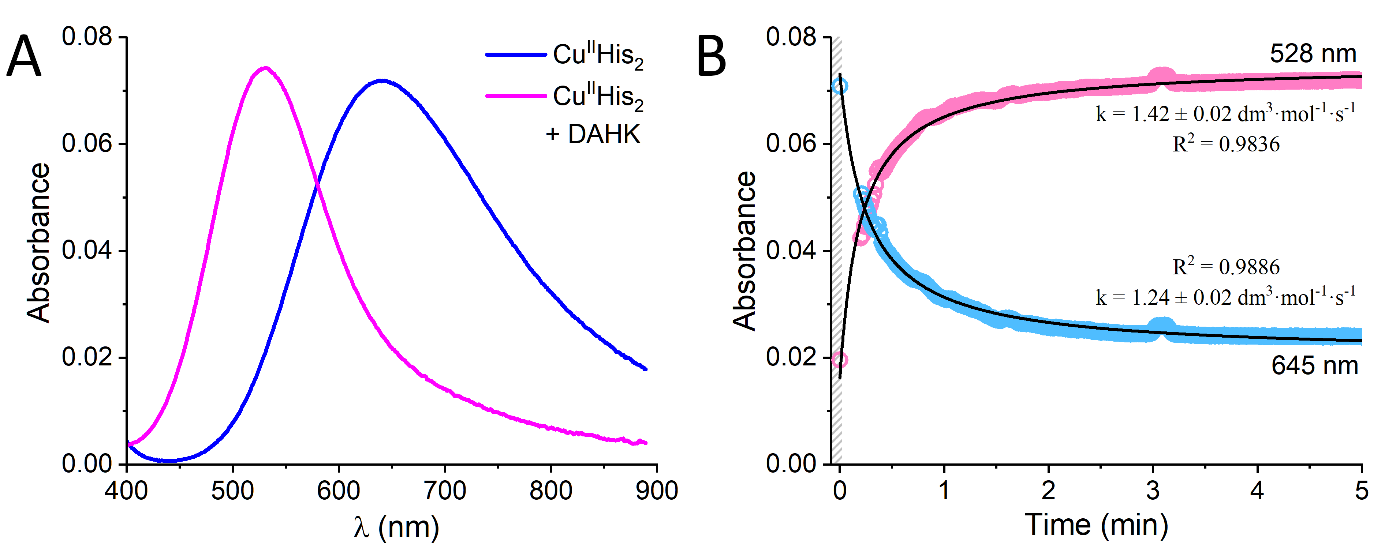


**Figure S9.** (A) Comparison of the absorption spectra obtained under equilibrium for 2 mM His in a presence of 0.9 mM CuCl_2_ before (blue line) and after (pink line) adding of 1 mM DAHK; (B) kinetic traces corresponding to Cu^II^ transfer from His (645 nm) to DAHK (528 nm) together with the second order fits, 200 mM HEPES, pH 7.4, 25°C. The experiment was performed on a classic UV-Vis spectrophotometer with temperature control (Cary 3500).


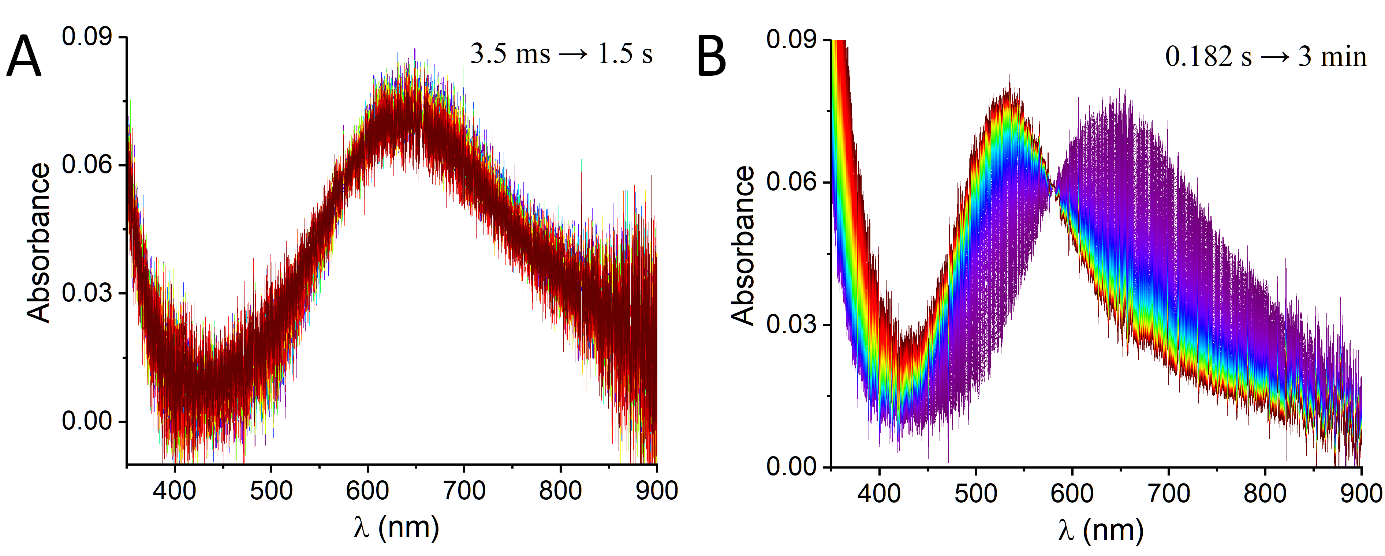


**Figure S10.** The absorption spectra collected for (A) 1.5 s with 1.5 ms intervals and (B) 3 min with 180 ms intervals, after mixing of 150 µL of 3 mM DAHK dissolved in 400 mM HEPES buffer with 150 µL of 2.7 mM CuCl_2_ dissolved in distilled water and 150 µL of 6 mM His dissolved in 200 mM HEPES, pH 7.4, 25°C. The experiment was performed in a three-syringe stopped-flow configuration (Figure S2B).


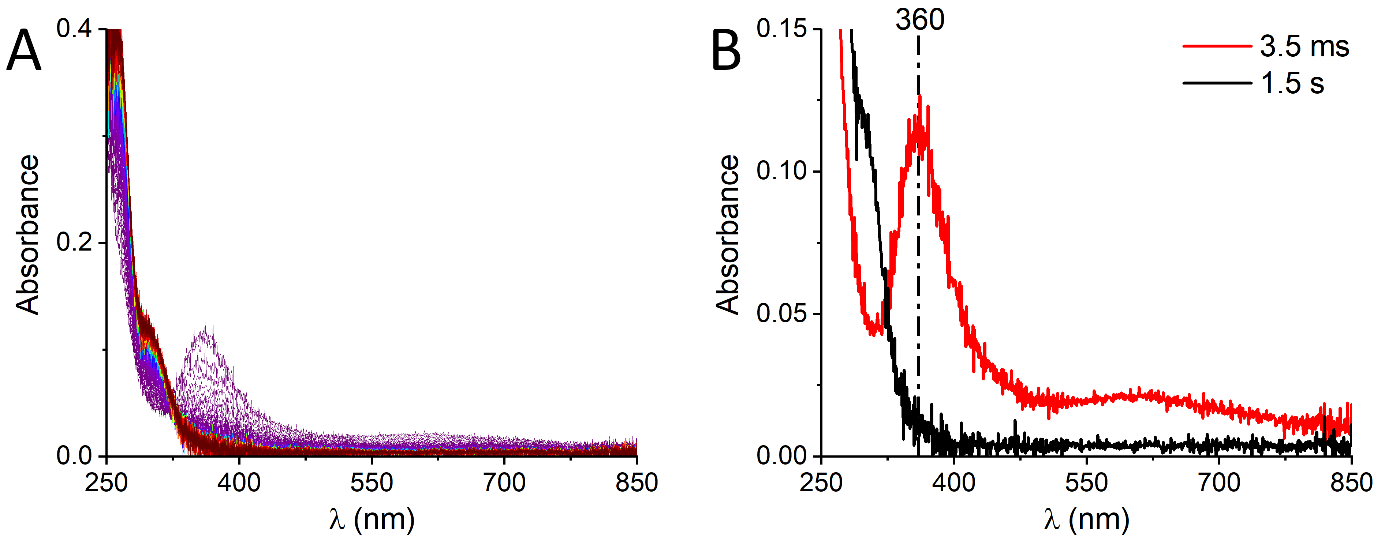


**Figure S11.** (A) The absorption spectra collected for 1.5 s with 1.5 ms intervals after mixing of 150 µL of 10 mM GSH dissolved in 400 mM HEPES with the same volume of 0.1 mM CuCl_2_ dissolved in distilled water, pH 7.4, 25°C; (B) comparison of the initial (3.5 ms, red line) and final (1.5 s, black line) absorption spectra. The experiment was performed in a two-syringe stopped-flow configuration (Figure S2A).


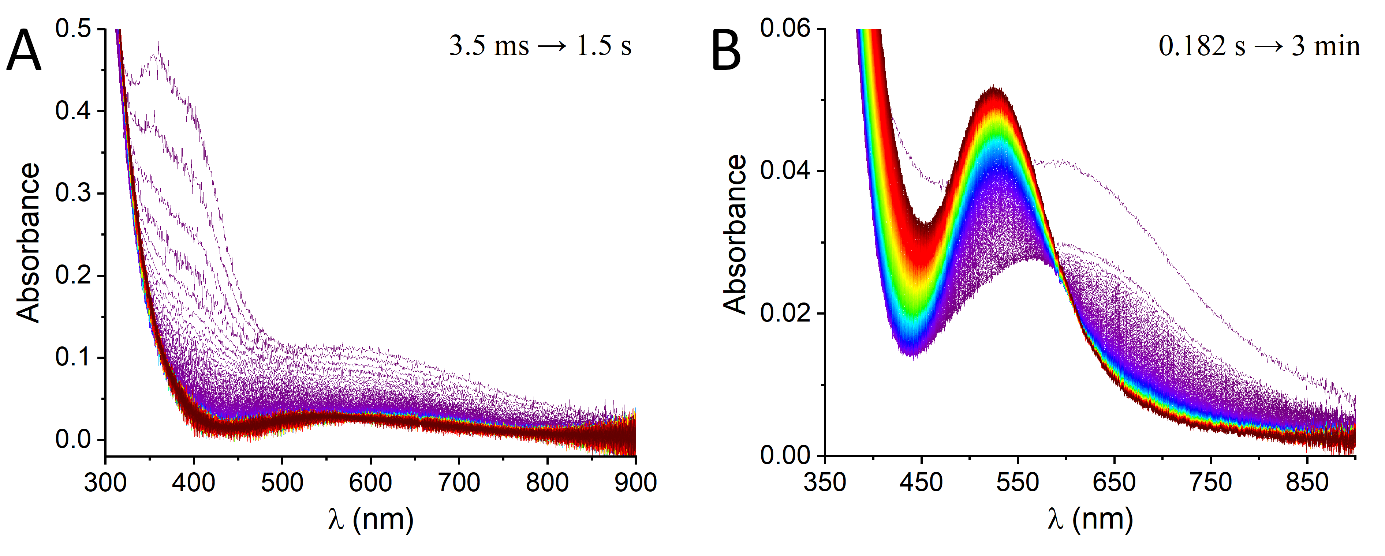


**Figure S12.** The absorption spectra collected for (A) 1.5 s with 1.5 ms intervals and (B) 3 min with 180 ms intervals, after mixing of 150 µL of 3 mM DAHK dissolved in 400 mM HEPES buffer with 150 µL of 2.7 mM CuCl_2_ dissolved in distilled water and 150 µL of 3 mM GSH dissolved in 200 mM HEPES, pH 7.4, 25°C. The experiment was performed in a three-syringe stopped-flow configuration (Figure S2B).


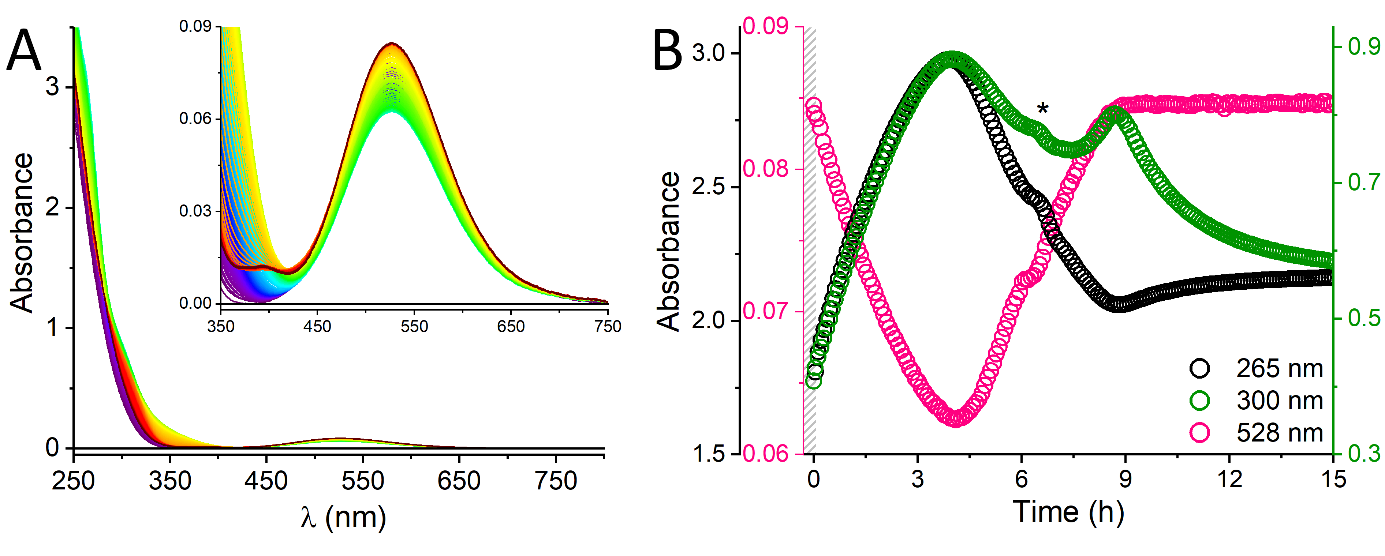


**Figure S13.** (A) The absorption spectra collected for 15 h with 5 min intervals for 1 mM DAHK with 0.9 mM CuCl_2_ in the presence of 1 mM GSH, (B) corresponding kinetic traces at selected wavelengths chosen according to the major intensity differences revealed by differential spectra (Figure S14); 200 mM HEPES, pH 7.4, 25°C.

* a disturbance observed at all wavelengths near 6 h (panel B) is an artefact arising from a technical issue of the spectrophotometer


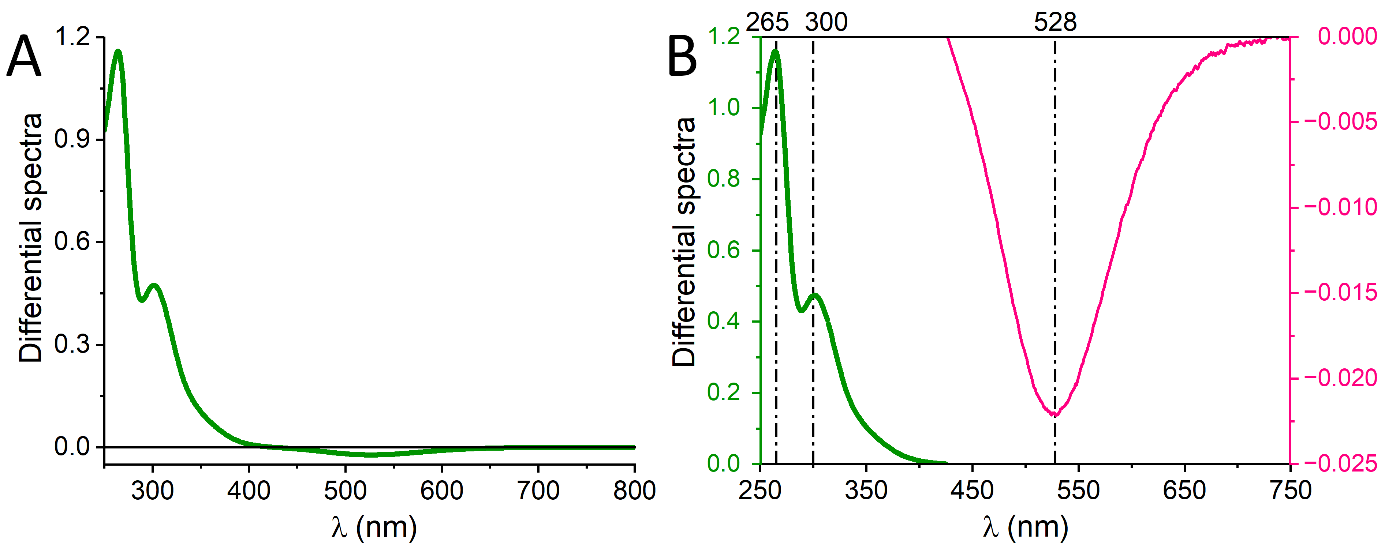


**Figure S14.** Differential absorption spectra obtained for the reaction of 1 mM DAHK with 0.9 mM CuCl_2_ in the presence of 1.0 mM GSH; panel (A) shows the entire spectral range in one scale, in panel (B) UV and Vis regions are shown separately in two different scales. The plot depicts regions of major changes occurring during the reduction step lasting around 4 h, expressed as a difference between 50^th^ cycle of the reaction and the Cu^II^DAHK initial spectrum; 200 mM HEPES, pH=7.4, T=25°C. The experiment was performed on a conventional UV-Vis spectrophotometer with temperature control (Cary 3500).
